# Spatial and temporal patterns of Ross River virus in south east Queensland, Australia: identification of hot spots at the rural-urban interface

**DOI:** 10.1186/s12879-020-05411-x

**Published:** 2020-10-02

**Authors:** Amanda K. Murphy, Julie A. Clennon, Gonzalo Vazquez-Prokopec, Cassie C. Jansen, Francesca D. Frentiu, Louise M. Hafner, Wenbiao Hu, Gregor J. Devine

**Affiliations:** 1 Mosquito Control Laboratory, QIMR Berghofer Medical Research Institute, Brisbane, Australia; 2 School of Biomedical Sciences, Faculty of Health, and Institute for Health and Biomedical Innovation, Queensland University of Technology, Brisbane, Australia; 3 Department of Biostatistics and Bioinformatics, Rollins School of Public Health, Emory University, Atlanta, USA; 4 Department of Environmental Sciences, Emory University, Atlanta, USA; 5 Communicable Diseases Branch, Queensland Health, Herston, Australia; 6 School of Public Health and Social Work, Queensland University of Technology, Brisbane, Australia

**Keywords:** Ross River virus, Arbovirus, Urban, Spatial, Epidemic, Queensland

## Abstract

**Background:**

Ross River virus (RRV) is responsible for the most common vector-borne disease of humans reported in Australia. The virus circulates in enzootic cycles between multiple species of mosquitoes, wildlife reservoir hosts and humans. Public health concern about RRV is increasing due to rising incidence rates in Australian urban centres, along with increased circulation in Pacific Island countries. Australia experienced its largest recorded outbreak of 9544 cases in 2015, with the majority reported from south east Queensland (SEQ). This study examined potential links between disease patterns and transmission pathways of RRV.

**Methods:**

The spatial and temporal distribution of notified RRV cases, and associated epidemiological features in SEQ, were analysed for the period 2001–2016. This included fine-scale analysis of disease patterns across the suburbs of the capital city of Brisbane, and those of 8 adjacent Local Government Areas, and host spot analyses to identify locations with significantly high incidence.

**Results:**

The mean annual incidence rate for the region was 41/100,000 with a consistent seasonal peak in cases between February and May. The highest RRV incidence was in adults aged from 30 to 64 years (mean incidence rate: 59/100,000), and females had higher incidence rates than males (mean incidence rates: 44/100,000 and 34/100,000, respectively). Spatial patterns of disease were heterogeneous between years, and there was a wide distribution of disease across both urban and rural areas of SEQ. Overall, the highest incidence rates were reported from predominantly rural suburbs to the north of Brisbane City, with significant hot spots located in peri-urban suburbs where residential, agricultural and conserved natural land use types intersect.

**Conclusions:**

Although RRV is endemic across all of SEQ, transmission is most concentrated in areas where urban and peri-urban environments intersect. The drivers of RRV transmission across rural-urban landscapes should be prioritised for further investigation, including identification of specific vectors and hosts that mediate human spillover.

## Background

Ross River virus (RRV) is a zoonotic alphavirus commonly circulating in Australia and the Western Pacific, and is responsible for the most widespread and frequently reported mosquito-borne disease in Australia [1, 2]. An average of 5409 RRV notifications were reported across Australia each year between 2006 and 2015 – an increase of 31% compared to the previous decade [1]. In humans, symptoms of infection are similar to those of other alphaviruses, such as Barmah Forest, chikungunya and Sindbis, and may include fever, rash, fatigue and polyarthritic muscle and joint pains [3]. While not fatal, RRV disease is associated with substantial morbidity and public health impact [4, 5], with symptoms including persistent pain and lethargy for weeks to months following infection [6–8]. Because there are no specific treatments for RRV, symptoms are managed through use of analgesic and anti-inflammatory drugs. It is estimated that notified cases represent only a small proportion of the infected population, as up to 80% of infections may be asymptomatic [9]. Although a vaccine has been developed, challenges in determining commercial viability have hindered its progress to market [5, 10].

Ross River virus cases are reported across all Australian states, although Queensland typically reports around half of all annual cases (average of 48%, ranging between 24 and 65% during 2001–2016). Historically considered a rural disease, cases have increasingly been observed in metropolitan areas of Perth, Brisbane, Sydney and Melbourne since the 1990s [11–16]. The largest recorded Australian RRV epidemic occurred from late 2014, and continued through 2015, culminating in a record annual total of 9544 cases in 2015 [1, 17]. Sixty-five percent of these cases (6193) were from Queensland, with 4388 reported from the capital, Brisbane – a five-fold increase compared with the previous 4 years [18]. Subsequent large outbreaks occurred in the states of New South Wales, Victoria and Western Australia in 2017, with the national total reaching 6928 cases [1]. These coincided with reports of unexpectedly high seroprevalence rates in Australia’s neighbouring Pacific Island countries [2, 19]. Although sporadic outbreaks in the Pacific Islands had been documented, these recent studies revealed that RRV circulates more regularly outside of Australia than previously thought [20].

RRV prevention relies solely upon mosquito control and avoidance of bites. Development of targeted strategies for RRV management is complicated by uncertainty about the specific vector and reservoir host species that are responsible for mediating epidemics [11, 21]. RRV is maintained in enzootic cycles between multiple species of mosquitoes, animal reservoir hosts and humans, but transmission pathways are poorly understood [22–24]. The virus infects several vertebrate host and mosquito vector species across different habitats and climate regions of Australia [3, 11]. This includes at least 40 different mosquito species, associated with both freshwater and saltwater habitats [11]. In coastal areas, the estuarine species *Aedes (Ae.) vigilax* (in northern Australia) and *Ae. camptohynchus* (in southern Australia) are considered likely vectors, while major inland vector species include freshwater-breeding *Culex (Cx.) annulirostris*, *Ae. procax* and the urban-associated *Ae. notoscriptus* [3]. Many RRV vector species also have diverse host feeding behaviours which likely vary with environmental setting [25].

Vertebrate host species that maintain RRV circulation also vary with einvironmental setting. In rural areas, marsupial mammals are suspected to be a major, but not sole, reservoir [20, 21]. Hosts such as humans, livestock, rodents and birds could also play a role in virus maintenance and amplification, particularly in urban areas where marsupials are less common [20]. However, the specific determinants of transmission, including the most important mosquito vectors and reservoir hosts in different habitat types and geographic locations, remain unknown [22–24]. Factors such as climate, vegetation cover, and human behaviour likely play a role in transmission, but their relative roles are not very well defined [26–30]. This diversity in the transmission cycle complicates epidemiological investigation, prediction and control [15, 22]. Hence, to be most informative, RRV studies require regional (rather than national-scale) approaches, adapted to specific ecological settings [31].

The south east Queensland (SEQ) region, including the capital city of Brisbane, experiences regular outbreaks and high morbidity caused by RRV. Despite this, few spatial and temporal analyses of RRV trends have been conducted over the past 2 decades, and none that assess the entire region. RRV is known to have been transmitted across both urban and peri-urban areas of Brisbane since at least the 1990’s, although the specific determinants of outbreaks remain uncertain [28, 29]. Studies of two large outbreaks in Brisbane indicated a wide distribution of cases across the city [14, 17], although increased risk has also been associated with living in proximity to freshwater bushland and wetland environments [29, 32]. We explored the contemporary distribution and epidemiological characteristics of RRV in SEQ between 2001 and 2016. Fine-scale spatial and temporal trends in distribution of RRV were assessed in urban and rural areas, with the aim to provide a detailed analysis of disease trends, and explore potential links between disease patterns and transmission pathways of RRV.

## Methods

### Study location

**Fig. 1:**
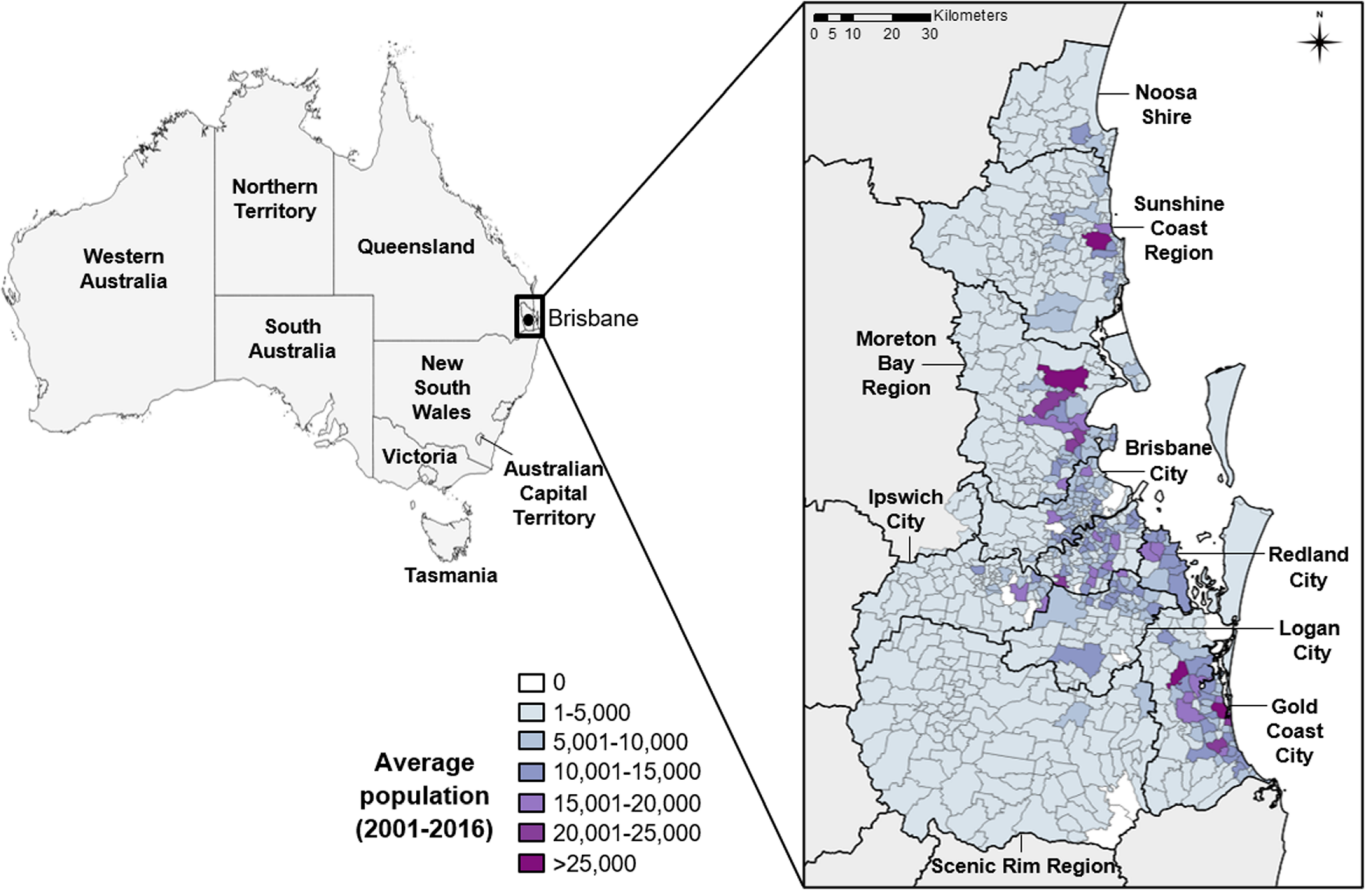
Map of Australia and south east Queensland. On left, the 8 Australian states are shown including the capital city of Brisbane. Inset, south east Queensland, and the 9 local government areas (LGAs) included in the study, each sub-divided into smaller State Suburb Code (SSC) units. The population distribution for SSCs is indicated by graduated shading

Queensland is Australia’s third most populated state, with 4.9 million inhabitants. We explored patterns of notified RRV cases in nine Local Government Areas (LGAs) of south east Queensland (SEQ): City of Brisbane, City of Ipswich, Moreton Bay Region, Redland City, Logan City, City of Gold Coast, Scenic Rim Region, Sunshine Coast Region and Shire of Noosa (Fig. 1). These 9 LGAs encompass an area of approximately 22,000 km^2^ and together comprise 80% of the state’s population (3.2 million), including 1.1 million in the capital city of Brisbane. The region has a sub-tropical climate, and diverse natural ecosystems including freshwater and estuarine wetlands, Mangrove shrubland and saltmarsh, Eucalypt and Melaleuca woodlands, and rainforest [33].

### RRV notification data

Daily RRV notification data were obtained from the Queensland Department of Health’s Notifiable Conditions Surveillance System (NoCS) for the 16-year period between 1st January 2001 and 31st December 2016. Case data included: disease onset date (an estimate, based on reported onset of illness at the time of presentation to a medical clinic), age, gender, and geographical location of case residence. The residential addresses of cases were aggregated to two different geographical unit classifications used by State and Territory Local Government Departments, and described in the Australian Standard Geographical Classification [34]. These were the LGA and the State Suburb Code (SSC). The broadest scale unit was the LGA, which is equivalent to a large municipal area, with a population of up to 1,100,000 people (average of 40,000); while SSC represented the finest geographical unit size, equivalent to a suburb or neighbourhood, comprising populations up to 50,000 (average 1500). The study area included a total of 774 SSCs across 9 LGAs, of which 17 were unpopulated and excluded from the analyses. Population data for LGAs and SSCs were extracted from census data from the Australian Bureau of Statistics (ABS) for census years between 2001 and 2016 [35]. Annual population figures, and population by gender and age-group, were matched to the notification data for each LGA and SSC for the calculation of incidence rates.

### Rural and urban classifications

State Suburb Codes were classified as rural or urban according to the Australian Statistical Geography Standard (ASGS), which defines urban areas of Australia [36]. The ASGS designates urban or rural categories based on population density and urban infrastructure criteria for each Section of State, obtained from census data. These were matched to each SSC using Geographical Information System (GIS) software ArcMap 10.6 (ESRI, Redlands, CA, USA). Urban areas are grouped into the sub-categories: ‘Major Urban’; a combination of Urban Centres with a total population of 100,000 or more, and ‘Other Urban’; a combination of Urban Centres with a population between 1000 and 99,999. Rural areas also have two sub-categories: ‘Bounded Locality’; a population centre of between 200 and 999 residents, and ‘Rural Balance’; which forms the Remainder of the State/Territory. The ABS considers both categories ‘Major Urban’ and ‘Other Urban’ as urban, while ‘Bounded Locality’ and ‘Rural Balance’ are rural. In our dataset, 292 SSCs were classified as rural and 465 as urban.

### Land use data

Land use maps were obtained from the Queensland Government Land Use Mapping Program (QLUMP), available from the Queensland Spatial Catalogue [37]. The QLUMP maps and assesses land use patterns and changes across the state, according to the Australian Land Use and Management (ALUM) Classification (Australian Department of Agriculture, version 8, October 2016) [38]. The ALUM Classification is a detailed national standard that classifies land use types in order of increasing levels of modification of the natural landscape. The 6 classes are: 1. Conservation and Natural Environments: Land is used primarily for conservation purposes, based on the maintenance of essentially natural ecosystems already present; 2. Production from Relatively Natural Environments: Land is used mainly for primary production based on limited change to the native vegetation; 3. Production from Dryland Agriculture and Plantations: Land is used mainly for primary production, based on dryland farming systems; 4. Production from Irrigated Agriculture and Plantations: Land is used mainly for primary production, based on irrigated farming; 5. Intensive uses: Land is subject to substantial modification, generally in association with closer residential settlement, commercial or industrial uses; and 6. Water. Land use maps were imported into ArcMap 10.6 (ESRI, Redlands, CA, USA) for visualisation.

### Data analysis

RRV notification epidemic features were explored over time, with monthly, annual and mean incidence rates calculated for LGAs, SSCs and the overall SEQ region. Spatial and temporal patterns of RRV notification rates were compared by age and sex, and by rural versus urban classification. Differences were tested for statistical significance using the Kruskal-Wallis or Mann-Whitney non-parametric tests, implemented in Statistical Package for the Social Sciences (SPSS) Statistics software (IBM New York USA; version 23), with a significance level of 0.05.

Spatial analyses included calculation of smoothed incidence rates and hot spot analysis, both performed using GeoDa software (Luc Anselin, version 1.12.1.161, September 2018). Spatial smoothing of rates were performed using Spatial Empirical Bayes (EB) smoothing technique, which corrects for outliers in raw (crude) rates to increase precision, especially where raw rates are unstable or have a high variance. High variances were present in the SEQ incidence data due to low case numbers being reported from some SSCs with small populations. These small population numbers can have the effect of skewing incidence rates to be extremely high. Incidence calculations and spatial analyses were performed using both raw rates and EB-smoothed rates to account for the effect that high variability in SSC population sizes had on incidence calculations. The Spatial EB technique recalculates the incidence rate for each geographical unit (SSC), applying an adjustment factor to each that is calculated relative to its raw rate and that of its immediate neighbouring geographical units (queen continguity scheme) [39]. This has the effect of reducing the rates of extremely high-rate SSCs, while also increasing the rate of low-rate SSCs, so that rates for each SSC become closer to that of their neighbours. Adjustment factors calculated for each SSC are also proportional to the population at risk, so that smaller populations will have their rates adjusted considerably, whereas rates for larger populations will change less. This is because larger populations provide greater confidence in the accuracy of the rates measured.

Hot spot analyses employed the Getis and Ord for G* local spatial autocorrelation statistic [40] for detecting hot and cold spot SSCs. Hot spot analysis was conducted using both raw and smoothed annual and mean incidence rates for SSCs, using queen contiguity neighbourhood criteria (adjusting rates by the average of immediate neighbouring SSCs in any direction). We identified and reported hot and cold spots that were significant in the raw analysis, and those that were shared between raw and smoothed analyses. Because hot/cold spots identified using mean rates were heavily influenced by the large magnitude outbreaks in 2014 and 2015, we also identified hot and cold spots that were persistently detected across at least two individual years. For this, we counted how many years an individual SSC was identified as a hot/cold spot, and defined those present in at least 2/16 years as persistent. Hot spots were then mapped against urban/rural classification and land use types of SEQ. Maps of population distribution, incidence rates and hot spots were created using ArcMap 10.6 (ESRI, Redlands, CA, USA). Hot spot locations were overlaid onto land use maps in ArcMap to enable visual comparsions between the two.

## Results

### Demographic trends

**Fig. 2:**
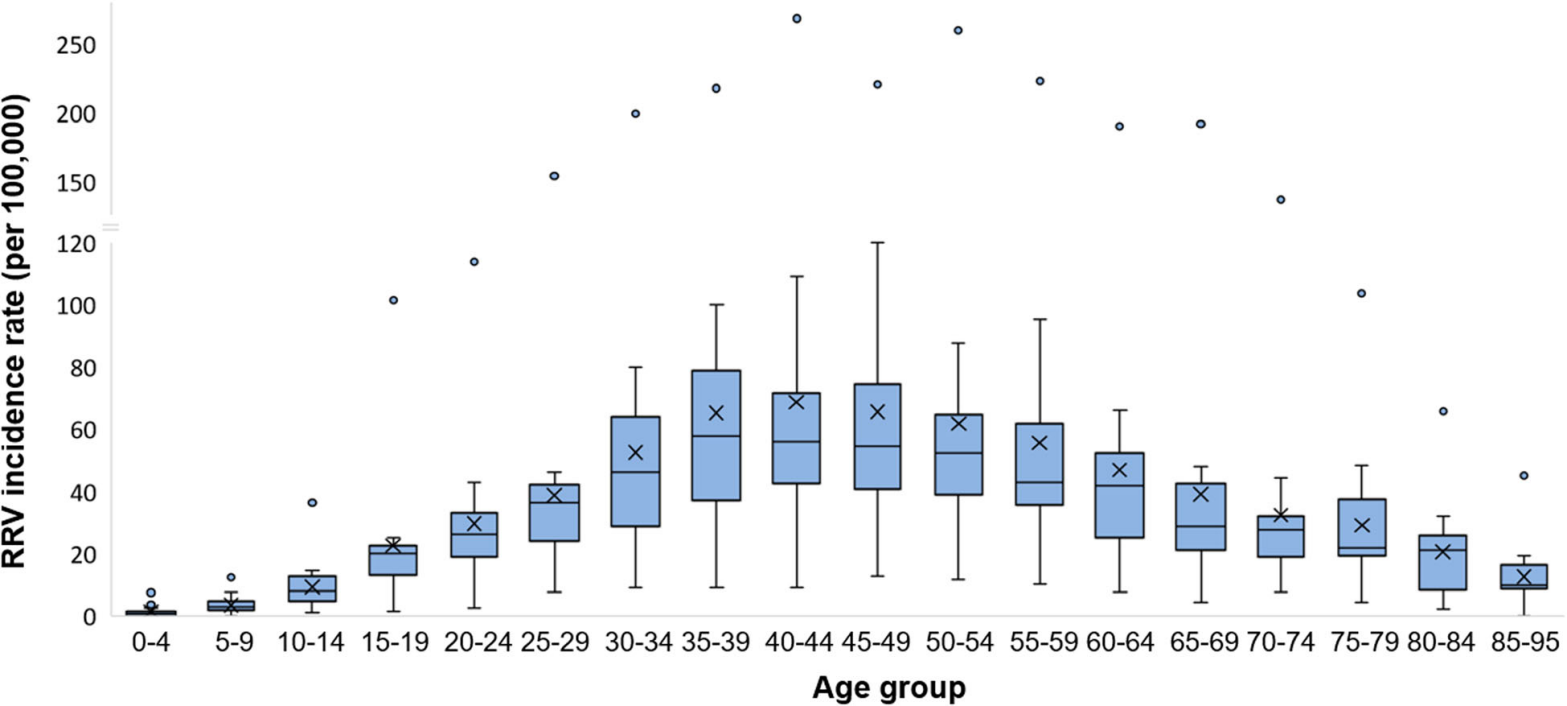
Mean annual RRV incidence in south east Queensland by age-group, 2001–2016. Age-adjusted mean annual incidence rates are grouped into 18 age groups of SEQ, between 0 and 95 years. Mean rates per group are indicated by X, and the median by the horizontal line across each box. Whiskers indicate the estimated minimum (lower whisker) and maximum (upper whisker) incidence values per age group (equal to the 1st and 3rd quartile −/+ 1.5 x the inter-quartile range, respectively). Outlying points indicate extreme values reported during large outbreak years, e.g. during the outbreaks of 2014 and 2015

For the period 2001–2016, a total of 18,115 RRV notifications were analysed across the SEQ region. During this period, the mean annual RRV notification rate across all of SEQ was 41 cases/100,000 population. Mean annual rates were higher in females versus males, at 44/100,000 population compared to 34/100,000, respectively, though this difference was not significant (Mann-Whitney U = 84, *p* = 0.102). For both genders, the highest incidence rates occurred in the 40–44 and 45–49 age categories (69 and 66/100,000, respectively) and the lowest in the two age categories < 10 years (1 and 3/100,000, respectively) (Fig. 2). The overall trend showed a gradual increase in incidence from birth up to age 29 years (mean incidence of 17/100,000 across these age groups), peaking between ages 30–64 years (mean 59/100,000), and dropping again ≥65 years (mean 27/100,000). This trend was consistent across all years of this study. Statistical comparisons within and between these three broad age groups indicated that rates did not differ significantly between adults from 30 to 64 years (Kruskal-Wallis H = 8, *p* = 0.239), but that this group’s rates were significantly higher than those aged ≤29 years (Mann-Whitney U = 1175, *p* < 0.0001), and ≥ 65 years (U = 1589, *p* < 0.0001).

### Temporal trends

**Fig. 3:**
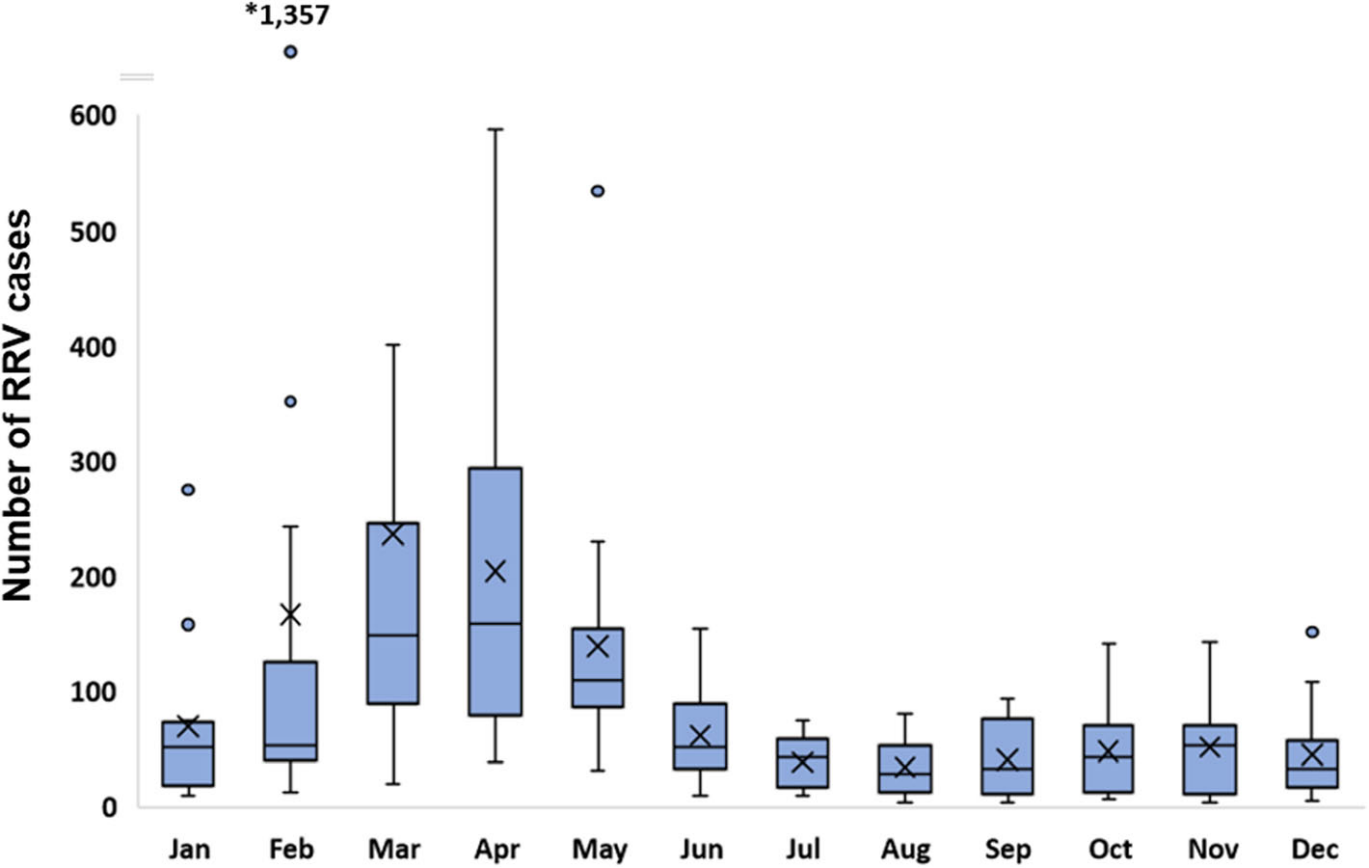
Mean monthly trend of RRV case notifications in south east Queensland, 2001–2016. Mean monthly case numbers across the 16-year period are shown, with extreme case report values from large outbreak years indicated as outliers. Mean rates per group are indicated by X, and median by the horizontal line crossing each box. Whiskers indicate the estimated maximum and minimum case numbers per month across the 16 years (equal to the 1st and 3rd quartile −/+ 1.5 x the inter-quartile range, respectively). *Total cases reported in February 2015 (1357) were the highest ever reported in a single month

RRV disease occured in SEQ throughout all months of the year; however, a distinct seasonal pattern in the timing of annual notification peaks was shared amongst all LGAs. The peak annual notification period was typically between February and May, and this was consistent across years with higher and lower notifications (Fig. 3). These months also showed the highest variability in case numbers (with a monthly average of 186 cases across SEQ, SD 246). Conversely, notifications during the winter and spring months between June–November were generally low and relatively stable across the region (monthly average of 46 cases, SD 35). The relative magnitude of RRV outbreaks across the region were variable between years, with larger and smaller outbreaks occurring in intermittent years (the monthly temporal trend is shown in Additional file 1). Long-term trends indicated that outbreaks generally occurred synchronously across the region, rather than initially occurring in one LGA and then spreading to another. This was also the case during largest recorded outbreak of 2015, which began earlier than usual (in late 2014) and peaked in February–March 2015, with timing consistent across all SEQ LGAs.

**Fig. 4:**
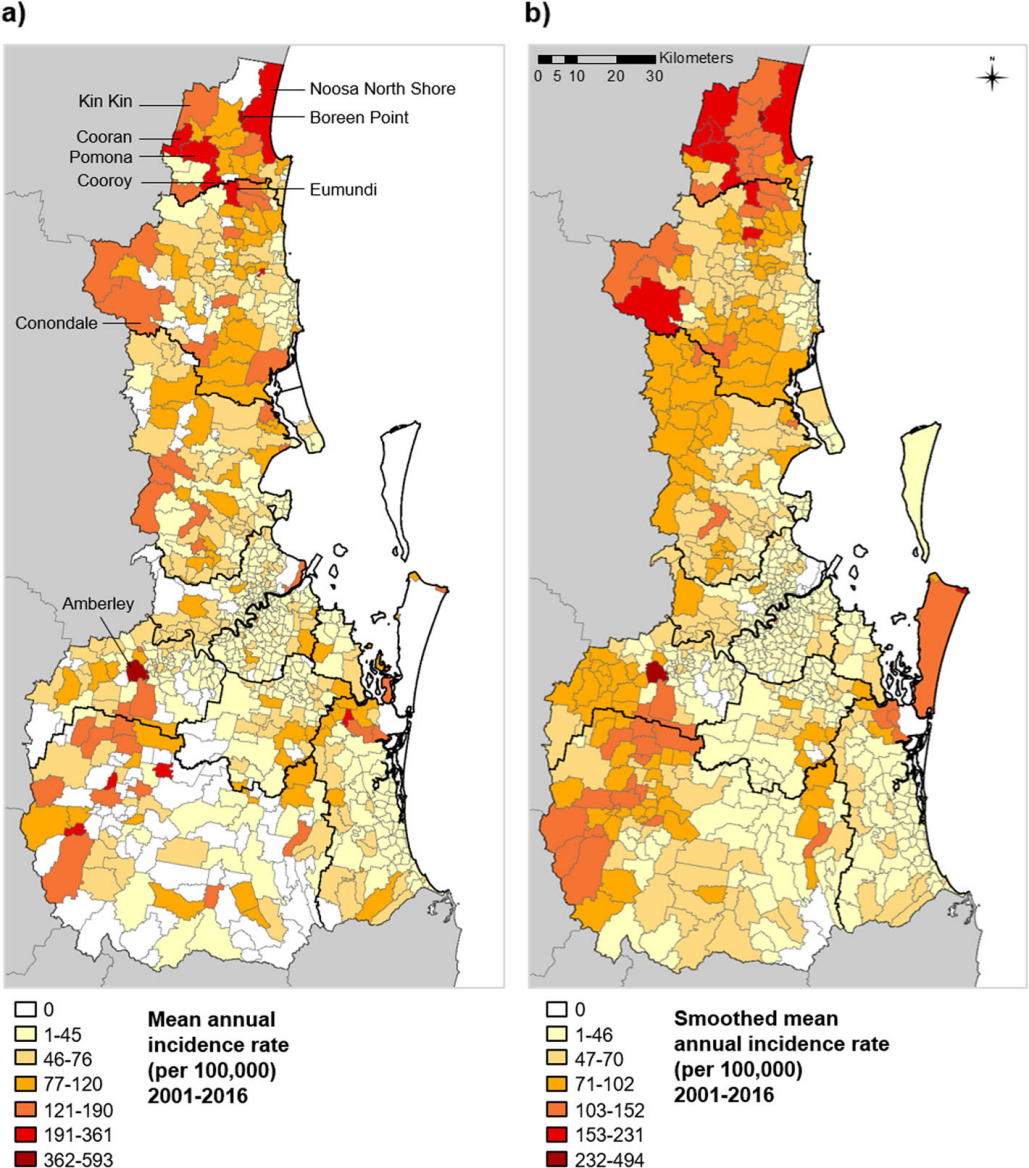
Mean annual RRV incidence of State Suburb Codes (SSCs) in south east Queensland, 2001–2016. **a** Mean annual incidence rate, and **b** smoothed mean annual incidence rate for SSCs of south east Queensland over the 16-year study period. Seventeen unpopulated SSCs are indicated by the white (zero case) locations in b)

**Fig. 5:**
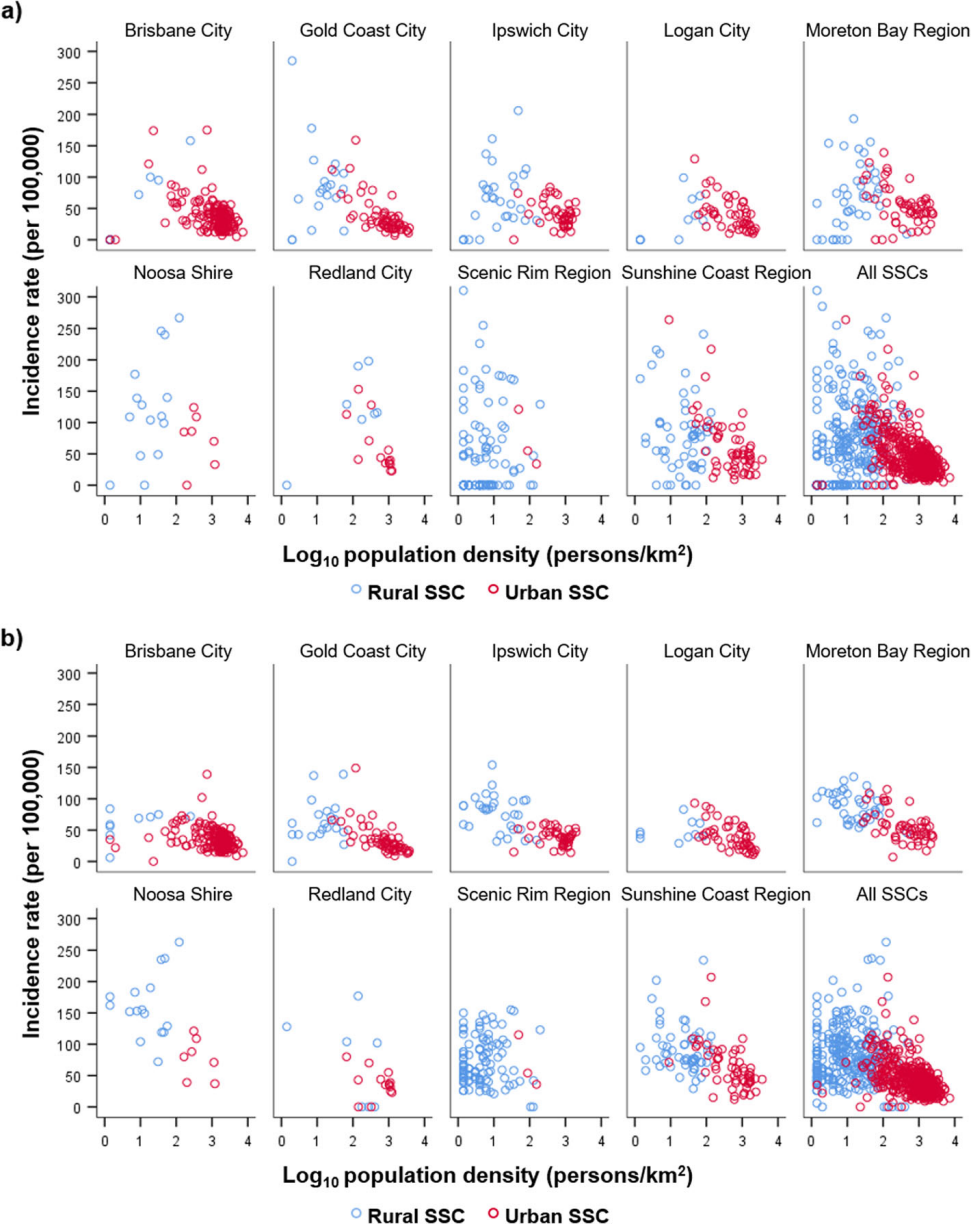
Mean annual RRV incidence of rural and urban State Suburb Codes (SSCs), 2001–2016. **a** raw incidence rates, and **b** smoothed incidence rates for the 757 SSCs of south east Queensland, shown according to the 9 Local Government Areas (LGAs) they are located within, and all SSCs combined. Rural and urban SSCs are indicated by blue and red circles, respectively. Two outlying SSCs (in Ipswich City and Noosa Shire) had incidence values beyond the scale shown here, but are listed in Additional file 4. See Fig. 1 for location of each LGA within the study area

### Spatial trends

Spatiotemporal patterns of RRV disease varied between the 9 LGAs, with the highest case numbers in the most populated LGA, Brisbane City (5352 total cases), and the lowest in the sparsely populated Scenic Rim Region (393 total cases) (Table 1). Annual case numbers for each LGA can be found in Additional file 2. Mean annual incidence rates across the 16-year period varied from 30/100,000 in Gold Coast City up to 130/100,000 in Noosa Shire (Table 1), with rates in Noosa Shire being significantly higher than all other LGAs (Kruskal Wallis H = 50, *p* < 0.0001; Mann-Whitney U = 58, *p* = 0.007 for pairwise comparison between Noosa Shire and Scenic Rim LGAs). Rates were also routinely high in the Sunshine Coast Region and sporadically high in the Scenic Rim Region. These 3 areas (Noosa Shire, Scenic Rim Region and Sunshine Coast Region) had the highest incidence rates overall, and had more rural characteristics (lower population density and proportion of urban SSCs) compared with the 4 largest cities: Brisbane, Gold Coast, Ipswich and Logan (Table 1). These 4 higher-density cities had the lowest rates of all LGAs.

At the SSC level, spatial trends were also highly varied. The spatial trend in high-incidence SSCs changed from one year to the next (recent annual patterns are shown in Additional file 3), but overall the SSCs with the highest raw and smoothed incidence rates were primarily located in Noosa Shire and the Sunshine Coast Region (Fig. 4, Additional file 4). However, the highest rate of all SSCs was for Amberley in Ipswich City LGA (raw mean rate 676/100,000 and smoothed mean rate of 562/100,000). The distribution of incidence rates for rural and urban SSCs in each LGA are shown in Fig. 5. The trend across LGAs showed that SSCs with the highest incidence rates tended to have low-mid range population densities. Mean annual RRV rates were 70 cases/100,000 (smoothed rate 85/100,000) in rural SSCs, and 44/100,000 (smoothed rate 43/100,000) in urban SSCs. Both raw and smoothed incidence rates were significantly higher in rural SSCs compared to urban SSCs (Mann-Whitney U=58,388, *p*=0.001 for raw; and U=24,359, *p*<0.0001 for smoothed).

**Table 1:** Summary characteristics of each Local Government Area (LGA) of south east Queensland during the study period, 2001–2016

LGA name^a^	Average population (2001–2016)	Population density (per km^2^)	Total cases (2001–2016)	Proportion of urban SSCs	Mean annual incidence rate (per 100,000)
Brisbane City	1,023,663	762	5352	94.2%	33
Gold Coast City	475,169	356	2252	74.1%	30
Ipswich City	157,963	146	1044	58.0%	41
Logan City	271,082	283	1456	81.7%	34
Moreton Bay Region	357,532	175	3097	58.5%	54
Noosa Shire	41,008	47	850	29.2%	130
Redland City	134,328	250	991	63.6%	46
Scenic Rim Region	35,443	8	393	2.8%	69
Sunshine Coast Region	256,982	114	2680	50.4%	65
**Total**	**2,753,170**	**188**	**18,115**	**61.0%**	**41**

^a^ See Fig. 1 for location of each LGA within the study area

### Hot spot analyses

Using raw mean annual incidence rates, a total of 127/757 (17%) SSCs were identified as hot spots and 352/757 (47%) as cold spots in any individual year. These increased to 178 and 458 SSCs, respectively, using smoothed rates. A comparison of raw versus smooth analyses for annual hot and cold spots across individual years was shown in Additional file 5. In either raw or smoothed analyses, 86 hot spots and 272 cold spots were persistent (present in ≥2 years). There were 14 SSCs that were both hot and cold spots in ≥2 years that were excluded, leaving 72 hot and 258 cold spots. Of these, 45 hot spots and 154 cold spots were identified as persistent in both raw and smooth analyses (shown in Additional file 6). Persistent hot spots were similar to those identified using mean annual rates (*n* = 56 mean hot spots), while mean cold spots differed (*n* = 47 mean cold spots). Although hot spots were geographically dispersed across all LGAs, the SSCs with the most persistent hot spots were in the Sunshine Coast Region and Noosa Shire LGAs. The same hot spots were rarely detected in consecutive years, although some were detected in multiple years, up to 7/16 years (Additional file 7). Conversely, cold spots tended to persist more in the same SSCs across several years, particularly in the Scenic Rim Region where there were very low populations and cases (Additional file 6).

**Fig. 6:**
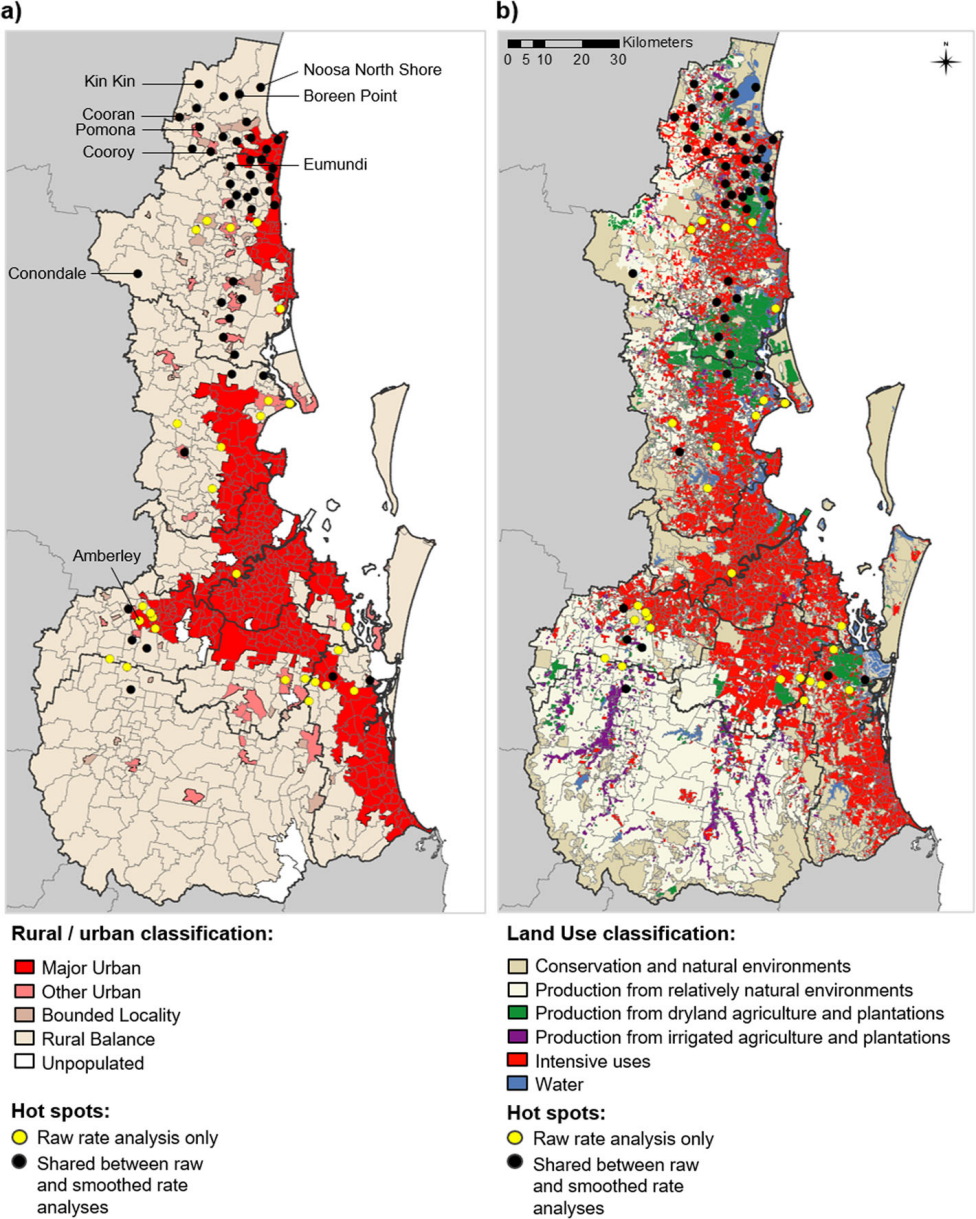
Persistent high incidence hot spots in south east Queensland, 2001–2016. Persistent hot spots identified for State Suburb Codes (SSCs) are shown relative to **a)** urban and rural areas of south east Queensland; and **b)** different land use types of south east Queensland. Points indicate the centroid of each hot spot SSC. Hot spots detected using raw incidence rates only (yellow points; *n* = 27 SSCs) and those detected in both raw and smoothed incidence analyses (black points; *n* = 45 SSCs) are indicated. Note: in b) ‘Intensive uses’ refers to residential areas and urban infrastructure. See methods for further description

A visualisation of hot spots relative to rural and urban areas of SEQ is shown in Fig. 6a. Hot spots tended to be most focused around the edges of where major urban and rural areas intersect. Of the 72 persistent hot spots detected in either raw or smoothed analyses, 35 were located in urban and 37 in rural SSCs (for the 45 hot spots shared between both analyses, 19 were urban and 26 rural). There also appeared to be diverse land use types within or adjacent to hot spot SSCs (Fig. 6b). All hot spots contained some degree of urban infrastructure, and many were located in close proximity to either dryland agriculture and plantations or to major water bodies. While some more inland hot spots were surrounded largely by conservation and natural environments, these environments appeared to be most often identified as cold spots (Fig. 6, Additional file 6). Very few hot spots were located centrally within major urban areas, rather than on the edge. Only one persistent hot spot SSC was identified within Brisbane City LGA (Chelmer, identified in 2/16 years) in the raw rate analysis only. The largest cities including Brisbane, Gold Coast and Logan LGAs were more commonly dominated by cold spots rather than hot spots (Additional files 5 and 6).

## Discussion

This study identified some epidemiological trends that may help elucidate the drivers of RRV spillover. Incidence rates were found to be highest for females, for age groups between 30 and 64 years, and in residents of rural suburbs (SSCs) north of Brisbane. Suburbs around the edges of major urban areas were persistent annual hot spots for RRV disease. This suggests that suburbs in rural and peri-urban areas possess characteristics that promote circulation of RRV, possibly related to specific habitat or land use types present. The specific contributors to human infection in different environments are uncertain, and this requires further investigation. In particular, identification of RRV vectors and hosts in areas where natural and urbanised environments meet.

The demographic trends observed were comparable with those of previous Australian studies, with the highest rates in females, and in age groups between 30 and 64 years [3, 8, 9, 11, 41]. Although male to female prevalence ratios have varied slightly in previous studies, no overall gender-related risk has been apparent [3, 8]. Clinical studies report that children show fewer symptoms than adults, presumably due to age-related differences in immune responses, while symptoms tend to persist longer in adults [8, 11, 41, 42]. However, seroprevalence studies have shown RRV antibody seroconversion to increase with age [43–45] suggesting that the true incidence of infection may be higher in younger age groups than indicated by notified cases. Therefore, higher notification rates in adults might reflect differing disease manifestations and treatment seeking practices with age, and not necessarily greater exposure to RRV. Demographic trends will also vary with geographical region, as the disease burden is higher in tropical northern regions of Australia compared to the temperate south [1]. Because few comprehensive seroprevalence studies have been conducted in Australia, the true age-related burden is unknown.

There was a strikingly consistent seasonal trend in outbreaks across SEQ, peaking annually between the months of February and May. This coincides with periods of relatively high temperature and rainfall from late austral summer to early autumn, when SEQ’s average daily temperatures are 20–24 °C [46]. Although climate alone does not predict outbreak occurrence, it influences vector and wildlife host species’ abundance [47–50]. Favourable temperature conditions, rainfall, high tides and low-level flooding have all been associated with elevated RRV risk in previous studies [26–28, 51–53]. Temperature also impacts viral replication, with the ideal temperature for RRV transmission at 26.4 degrees celsius (transmission range of 17 to 31.5 °C) [54]. However, variations in weather patterns and vector-host ecology mean that climate-based predictions are only valid locally or, at best, regionally, rather than nationally [55, 56]. Hence, although suitable weather conditions are a requisite precursor to outbreaks, outbreak occurrence ultimately depends on availability of, and interactions between, sufficient competent vectors and susceptible hosts [57, 58].

The observed variation in annual spatial trends suggests that conditions supporting transmission occur sporadically in particular SSCs, and may change from 1 year to the next. This might be due to local climatic and environmental variations which influence vector and host abundance in both freshwater and saltwater habitats [23, 27]. Freshwater vectors *Cx. annulirostris*, *Ae. notoscriptus*, *Ae. procax*, and *Ae. vittiger* as well as saltwater vectors *Ae. vigilax*, *Cx. sitiens* and *Verallina funerea* were associated with large outbreaks in Brisbane and the Sunshine Coast Region during the 1990s [14, 59]. The most recent outbreaks of 2014–2015 were linked to increased abundance of the freshwater vectors *Cx. annulirostris* and *Ae. procax* in Brisbane following high rainfall [17]. Many of the implicated vectors share similar or overlapping habitat types and have broad host-feeding behaviours [25]. It is possible that multiple vectors in different habitats contribute to varying degrees, at different times [60, 61].

Similarly, a number of different hosts that maintain RRV circulation across SEQ could contribute to epidemics. Although few studies have investigated the role of specific wildlife hosts in human RRV outbreaks, opportunistic serosurveys of wildlife together with a handful of experimental infection studies have generated some hypotheses [21]. Potential hosts theorised to contribute to RRV transmission include birds, small mammals and marsupials (including rodents, possums, flying foxes) in urban areas; and larger mammals and marsupial macropods (such as horses and cattle, kangaroos and wallabies) in peri-urban and rural areas [15, 20, 21, 57]. However, current evidence identifying important RRV hosts is limited, and broader investigations of the transmission potential of wildlife are much needed [57]. In the absence of these, it can be assumed from RRV’s wide geographic and habitat range that there is flexibility in both vectors and hosts. In SEQ, the seasonal composition of vectors and hosts in peri-urban habitats, especially those in proximity to hot spot suburbs, should be a particular focus for future RRV transmission studies.

The study identified both high incidence rates and the most persistent hot spots overall in Noosa Shire and Sunshine Coast Region, where there are low-medium human population densities and diverse land use types present. The specific drivers of high rates of RRV in these LGAs are uncertain, but could relate to the proximity of peri-urban human populations to rural vector and wildlife habitats. Interactions between humans, vectors and wildlife in or near particular land use types in peri-urban areas could create a ‘perfect storm’ of factors supporting RRV transmission. Human-modified and fragmented landscapes are known to influence the risk of vector borne diseases, either positively or negatively, through altering ecological relationships between wildlife, vectors and humans [62–64].. Land use changes such as deforestation and agricultural development have been linked to increased risk of West Nile virus and malaria infection [65, 66], and have been linked to arboviral disease risk in Australia [67, 68]. While this study’s findings do not confirm a link between land use and RRV risk, they do suggest this could be worthy of investigation. Given RRV’s expansion into urban and outer metropolitan areas of Australia in recent years, it is conceivable that urban expansion and alteration of wildlife habitats may have implications for RRV risk.

The absence of hot spots, and concentration of cold spots, in the Scenic Rim LGA suggests that it lacks sufficient human, wildlife host and vector populations to maintain persistent outbreaks. This is despite the Scenic Rim having a large proportion of natural conservation and irrigated agricultural areas, which could theoretically support mosquito and wildlife habitats. This might be explained by the low human population in this LGA, which results in sporadically high but inconsistent incidence rates, and unstable spatial patterns of disease (both hot and cold spots in the same suburbs in different years). This pattern could potentially change if human populations in the Scenic Rim were to increase. Again, analyses of different land use types, their association with specific vector and host habitats, and RRV risk would assist understanding of how and where these factors inter-relate.

This study is the first to describe long-term epidemiological trends of RRV across SEQ. RRV disease patterns were reported at both broad (LGA) and fine (SSC) scales, and characteristics associated with higher RRV risk were identified that can inform future investigations. However, the study was limited by its reliance on routinely collected public health data, and the associated challenges with passive disease reporting. In Queensland, notification processes for RRV do not include individual case interview, nor information on the timing and location of RRV exposure, which is often unknown. This study used the case’s reported onset date of symptoms and residential address as a proxy for this. While many residents will likely be bitten and infected in their home suburb, this will not be true for all, and there is no way to correct for this. Nevertheless, given RRV’s high incidence and wide geographical range across SEQ, using the case residence seems a reasonable proxy for location of infection. Socioecological factors not accounted for in our study may also have influenced the demographic trends observed. For instance, healthcare-seeking practices likely differ between genders and age-groups, and exposure to mosquitoes through occupational or leisure activities could also differ between demographic groups. The impact of socioeconomic factors on infection risk also varies by geographic region [22, 23, 28]. However, within the SEQ region, previous studies suggest that socioeconomic variation is unlikely to have had a significant impact on our results [28, 32].

## Conclusions

Overall, this study contributes to understanding of RRV disease patterns and public health risk in Australia. To further progress understanding of RRV transmission risk and improve future disease prediction and prevention, greater understanding of seasonal variation in distribution and abundance of potential vectors and hosts is essential. This study identified urban fringe areas and areas undergoing urban expansion as important risk factors for RRV transmission. Peri-urban suburbs at the rural-urban interface, especially where different land use types intersect, could be most capable of supporting adequate densities of vectors, hosts and humans to allow persistent transmission. It is recommended that hot spots identified in these ‘edge’ locations be targeted for further investigation of RRV transmission pathways. Clarifying the relative importance of specific contributors to RRV epidemics is a priority for developing targeted disease prevention strategies – and may have flow-on benefits for prevention of other endemic and imported mosquito-borne infections in Australia.

## Supplementary information

**Additional file 1: Figure S1.** Monthly trend of RRV notifications in south east Queensland, 2001–2016. Monthly case notifications are shown for each of the 9 Local Government Areas (LGAs) in the study area. A major flooding event that occurred in the region in early 2011 likely reduced case numbers of that year by inundating vector breeding sites with fast-flowing water. The largest ever recorded peak in monthly cases occurred for all LGAs during February and March 2015.

**Additional file 2: Table S2.** Summary of annual case counts for each Local Government Area (LGA).

**Additional file 3: Figure S3.** Annual RRV incidence in south east Queensland: 2013–2016. Annual incidence patterns are shown for State Suburb Codes (SSCs) within each of the 9 Local Government Areas in the years before, during and after the largest recorded epidemic in 2015.

**Additional file 4: Table S4.** Locations with the highest overall rates across all years, 2001–2016.

**Additional file 5: Figure S5.** Annual hot and cold spots for RRV incidence in south east Queensland: 2013–2016. Significant high- and low-incidence (hot and cold) spots identified through two different analysis techniques are overlaid: local G* analysis of raw (crude) annual incidence rates for State Suburb Codes (SSCs), and smoothed annual rates for SSCs (Empirical Bayes Spatial smoothing technique). Disagreement occurred where an SSC was hot in one analysis and cold in the other, or vice versa.

**Additional file 6: Figure S6.** Persistent and mean RRV hot spots in south east Queensland: 2001–2016. Significant high- and low-incidence (hot and cold) spots shared between raw and smoothed incidence rate analyses are shown by State Suburb Code (SSC): **a)** 45 persistent hot and 154 persistent cold spots (present in ≥2 years) present in both raw and smoothed analyses; and **b)** 56 mean hot and 47 mean cold spots present in both raw and smoothed analyses. In a) SSC colours are graduated according to the number of years identified as a hot/cold spot, including 14 additional SSCs that were both hot and cold in ≥2/16 years.

**Additional file 7: Table S7.** Summary of 45 persistent hot spots identified in both raw and smoothed incidence analyses from 2001 to 2016.

## Data Availability

All data generated or analysed during this study are included in this published article [and its supplementary information files].

## Abbreviations

ABS: Australian Bureau of Statistics
Ae.: Aedes
ALUM: Australian Land Use and Management
ASGS: Australian Statistical Geography Standard
Cx.: Culex
EB: Empirical Bayes
GIS: Geographical Information System
LGA: Local Government Area
NoCS: Notifiable Conditions Surveillance System
QIMRB: QIMR Berghofer Medical Research Institute
QLUMP: Queensland Government Land Use Mapping Program
QUT: Queensland University of Technology
RRV: Ross River virus
SEQ: south east Queensland
SSC: State Suburb Code
SPSS: Statistical Package for the Social Sciences
