# Supplementary figures and images for: Spatial and temporal patterns of Ross River virus in south east Queensland, Australia: identification of hot spots at the rural-urban interface
Source: BMC Infect Dis. 2020 Oct 2;20:722. doi: 10.1186/s12879-020-05411-x (PMC7530966; doi:10.1186/s12879-020-05411-x)

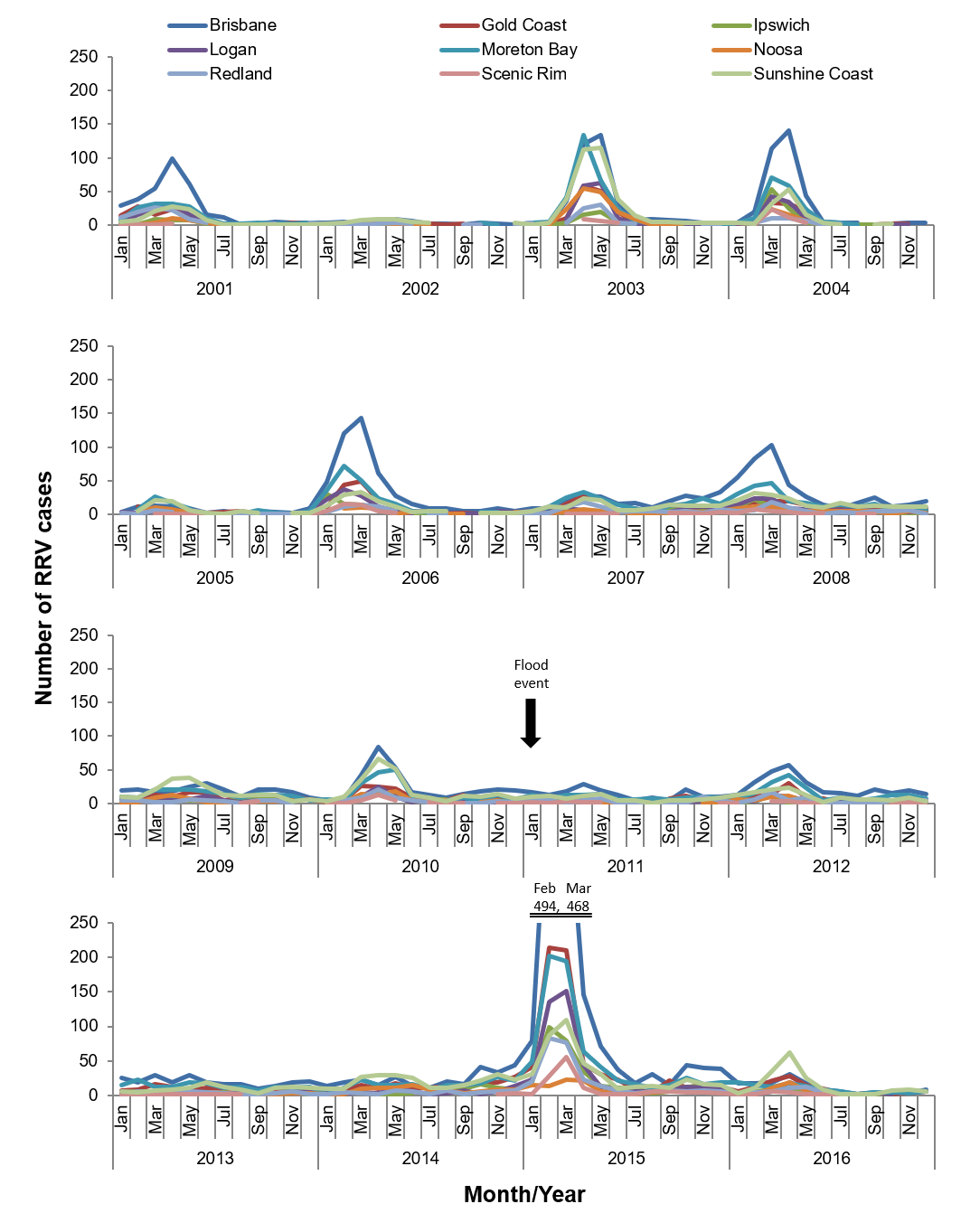

Supplement: Supplementary file 1 — Additional file 1: Figure S1. Monthly trend of RRV notifications in south east Queensland, 2001–2016. Monthly case notifications are shown for each of the 9 Local Government Areas (LGAs) in the study area. A major flooding event that occurred in the region in early 2011 likely reduced case numbers of that year by inundating vector breeding sites with fast-flowing water. The largest ever recorded peak in monthly cases occurred for all LGAs during February and March 2015. [file 12879_2020_5411_MOESM1_ESM.tif]

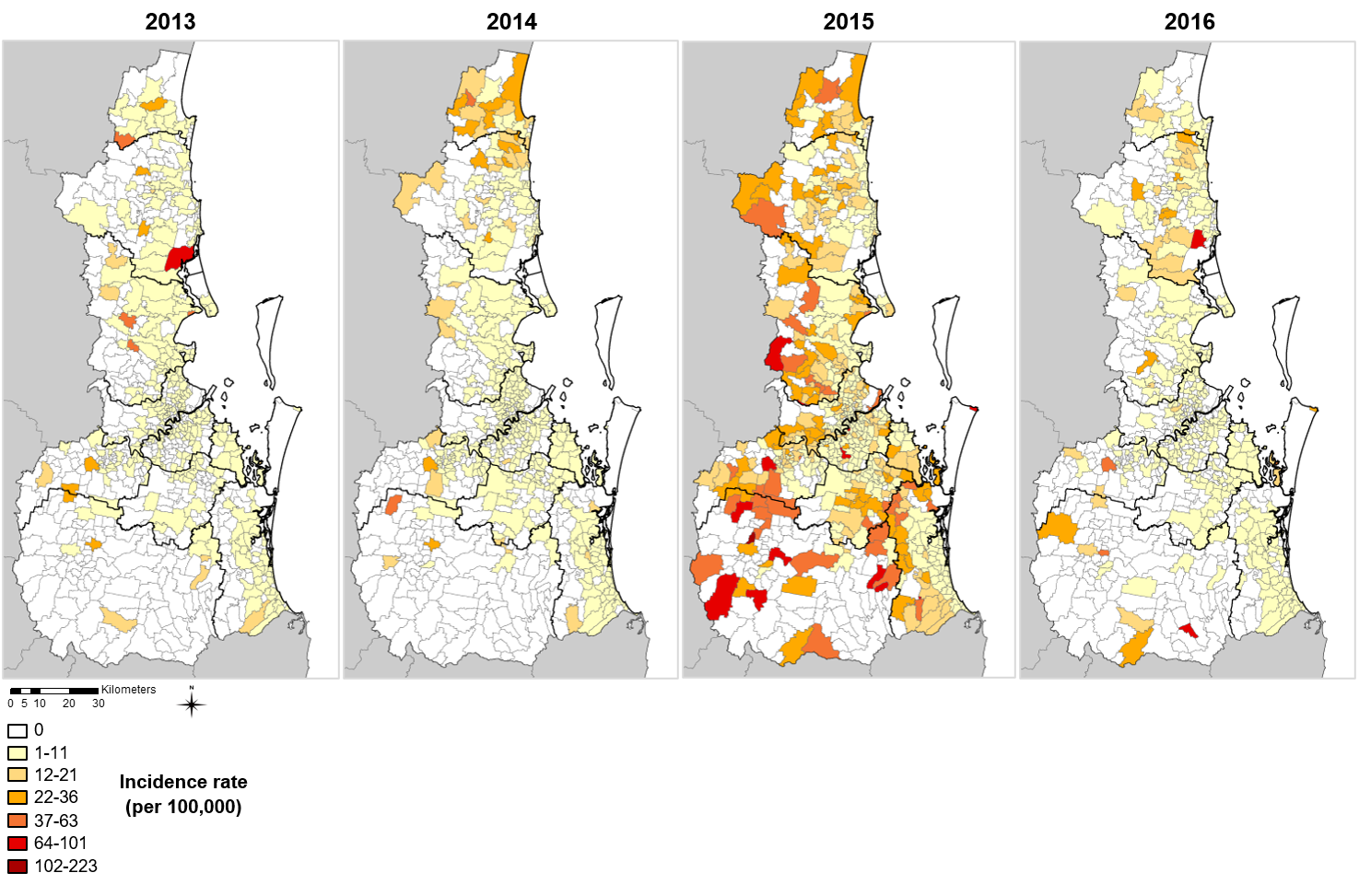

Supplement: Supplementary file 3 — Additional file 3: Figure S3. Annual RRV incidence in south east Queensland: 2013–2016. Annual incidence patterns are shown for State Suburb Codes (SSCs) within each of the 9 Local Government Areas in the years before, during and after the largest recorded epidemic in 2015. [file 12879_2020_5411_MOESM3_ESM.tif]

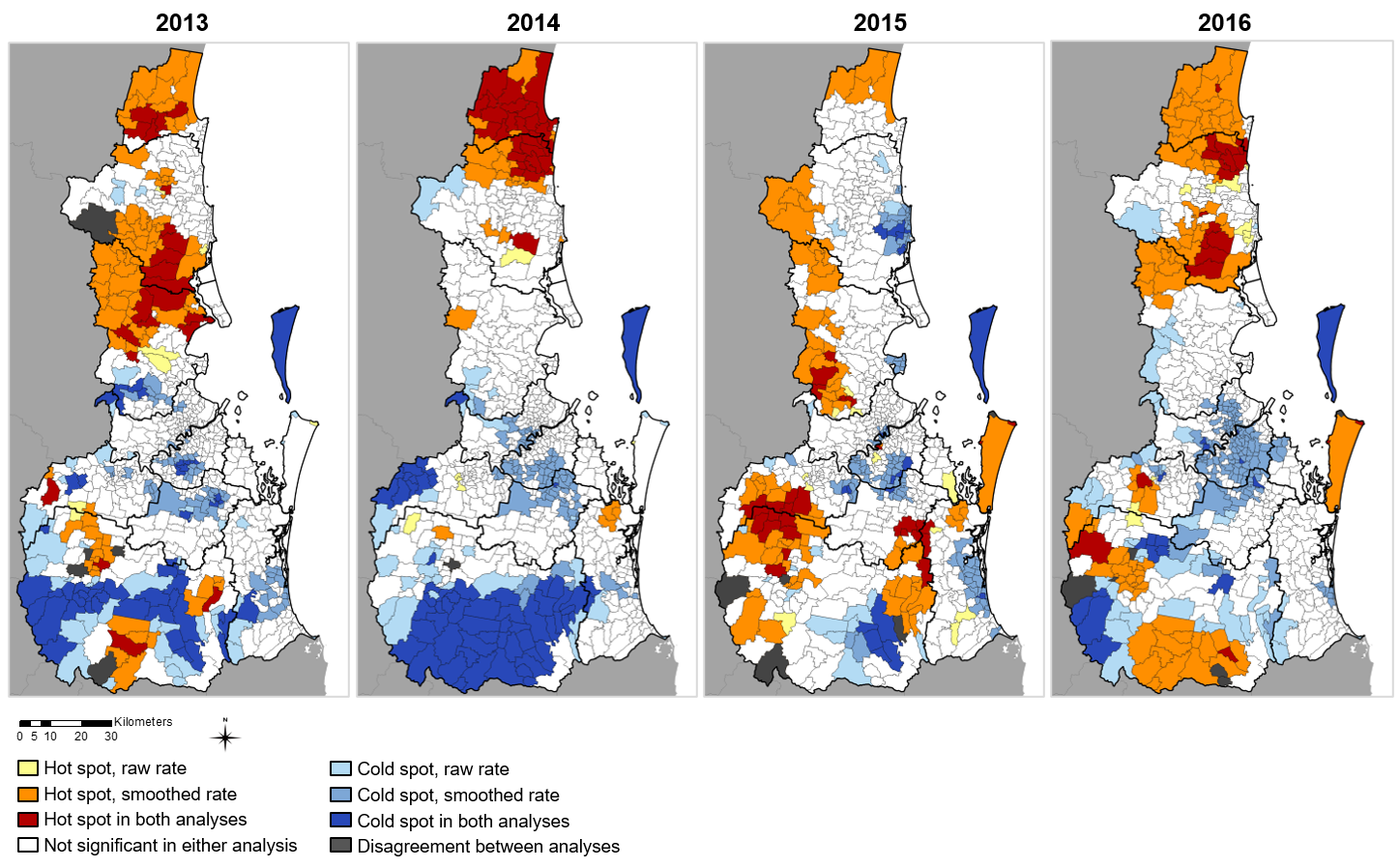

Supplement: Supplementary file 5 — Additional file 5: Figure S5. Annual hot and cold spots for RRV incidence in south east Queensland: 2013–2016. Significant high- and low-incidence (hot and cold) spots identified through two different analysis techniques are overlaid: local G* analysis of raw (crude) annual incidence rates for State Suburb Codes (SSCs), and smoothed annual rates for SSCs (Empirical Bayes Spatial smoothing technique). Disagreement occurred where an SSC was hot in one analysis and cold in the other, or vice versa. [file 12879_2020_5411_MOESM5_ESM.tif]

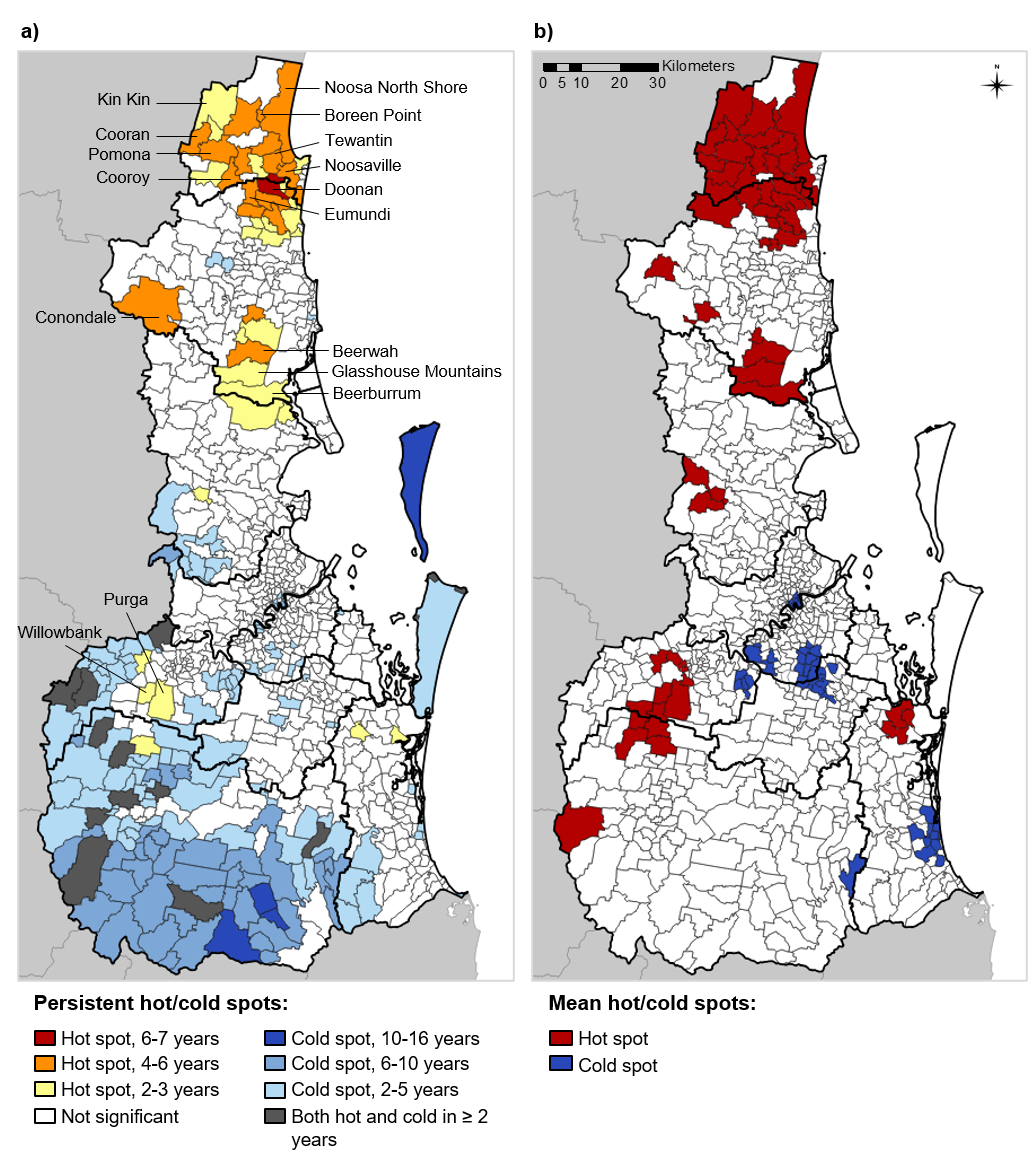

Supplement: Supplementary file 6 — Additional file 6: Figure S6. Persistent and mean RRV hot spots in south east Queensland: 2001–2016. Significant high- and low-incidence (hot and cold) spots shared between raw and smoothed incidence rate analyses are shown by State Suburb Code (SSC): a) 45 persistent hot and 154 persistent cold spots (present in ≥2 years) present in both raw and smoothed analyses; and b) 56 mean hot and 47 mean cold spots present in both raw and smoothed analyses. In a) SSC colours are graduated according to the number of years identified as a hot/cold spot, including 14 additional SSCs that were both hot and cold in ≥2/16 years. [file 12879_2020_5411_MOESM6_ESM.tif]
