# Supplementary material for: Spatial and temporal patterns of Ross River virus in south east Queensland, Australia: identification of hot spots at the rural-urban interface
Source: BMC Infect Dis. 2020 Oct 2;20:722. doi: 10.1186/s12879-020-05411-x (PMC7530966; doi:10.1186/s12879-020-05411-x)
Supplement: Supplementary file 2 — Additional file 2: Table S2. Summary of annual case counts for each Local Government Area (LGA). [file 12879_2020_5411_MOESM2_ESM.pdf]

Table S2. Summary of annual case counts for each Local Government Area (LGA).

LGA name	2001	2002	2003	2004	2005	2006	2007	2008	2009	2010	2011	2012	2013	2014	2015	2016	Grand Total
Brisbane City	325	39	363	342	74	454	239	418	224	299	168	292	231	272	1,477	135	**5,352**
Gold Coast City	106	21	153	99	37	153	125	138	121	122	61	102	107	164	636	107	**2,252**
Ipswich City	28	11	52	92	6	80	47	76	54	66	45	44	41	65	300	37	**1,044**
Logan City	95	11	145	99	24	117	68	111	60	60	30	59	48	59	424	46	**1,456**
Moreton Bay Region	150	26	289	176	73	212	190	229	160	186	112	175	160	182	672	105	**3,097**
Noosa Shire	29	12	161	48	26	35	36	63	46	68	15	44	34	85	103	45	**850**
Redland City	97	13	69	33	17	49	70	64	37	61	44	51	40	47	255	44	**991**
Scenic Rim Region	7	4	22	41	5	42	13	24	12	31	14	15	14	10	122	17	**393**
Sunshine Coast Region	98	41	342	111	64	123	143	205	195	234	89	123	109	236	399	168	**2,680**
**Total**	**935**	**178**	**1,596**	**1,041**	**326**	**1,265**	**931**	**1,328**	**909**	**1,127**	**578**	**905**	**784**	**1,120**	**4,388**	**704**	**18,115**
