# Supplementary material for: Spatial and temporal patterns of Ross River virus in south east Queensland, Australia: identification of hot spots at the rural-urban interface
Source: BMC Infect Dis. 2020 Oct 2;20:722. doi: 10.1186/s12879-020-05411-x (PMC7530966; doi:10.1186/s12879-020-05411-x)
Supplement: Supplementary file 4 — Additional file 4: Table S4. Locations with the highest overall rates across all years, 2001–2016. [file 12879_2020_5411_MOESM4_ESM.pdf]

**Table S4. Locations with the highest overall rates across all years, 2001-2016.**

| LGA name | SSC name | Urban/rural classification[1] | Average population[2] | Population density (persons/km$^2$) | Total cases (2001-2016) | Mean raw annual incidence rate* | Mean smoothed annual incidence rate* |
|---|---|---|---|---|---|---|---|
| Ipswich City | Amberley | Rural Balance | 222 | 8 | 24 | 676 | 562 |
| Noosa Shire | Boreen Point | Rural Balance | 287 | 55 | 19 | 413 | 363 |
| Noosa Shire | Noosa North Shore | Rural Balance | 157 | 1 | 9 | 359 | 176 |
| Scenic Rim Region | Woolooman | Rural Balance | 20 | 1 | 1 | 310 | 67 |
| Gold Coast City | Gilberton | Rural Balance | 22 | 2 | 1 | 285 | 61 |
| Noosa Shire | Cooroy | Rural Balance | 3,323 | 121 | 142 | 267 | 263 |
| Sunshine Coast Region | Kunda Park | Major Urban | 24 | 9 | 1 | 264 | 71 |
| Scenic Rim Region | Kents Lagoon | Rural Balance | 49 | 5 | 2 | 255 | 147 |
| Noosa Shire | Cooran | Rural Balance | 1,423 | 38 | 56 | 246 | 235 |
| Sunshine Coast Region | Eumundi | Rural Balance | 1,947 | 83 | 75 | 241 | 234 |
| Noosa Shire | Pomona | Rural Balance | 2,551 | 48 | 98 | 240 | 237 |
| Scenic Rim Region | Mount Edwards | Rural Balance | 55 | 4 | 2 | 226 | 125 |
| Sunshine Coast Region | Yandina | Other Urban | 2,078 | 136 | 72 | 217 | 207 |
| Sunshine Coast Region | Conondale | Rural Balance | 752 | 4 | 26 | 216 | 202 |
| Sunshine Coast Region | Elaman Creek | Rural Balance | 60 | 5 | 2 | 210 | 138 |
| Ipswich City | Blacksoil | Rural Balance | 91 | 48 | 3 | 206 | 74 |
| Redland City | Lamb Island | Bounded Locality | 379 | 273 | 12 | 198 | 0 |
| Moreton Bay Region | Samsonvale | Rural Balance | 517 | 15 | 16 | 193 | 135 |
| Sunshine Coast Region | Kenilworth | Rural Balance | 489 | 3 | 15 | 192 | 173 |
| Redland City | Point Lookout | Rural Balance | 625 | 139 | 19 | 190 | 177 |
| Scenic Rim Region | Mutdapilly | Rural Balance | 270 | 6 | 8 | 185 | 139 |
| Scenic Rim Region | Moogerah | Rural Balance | 205 | 1 | 6 | 183 | 123 |
| Sunshine Coast Region | Peachester | Rural Balance | 1,189 | 26 | 34 | 179 | 138 |
| Gold Coast City | Norwell | Rural Balance | 175 | 7 | 5 | 178 | 98 |

| LGA name | SSC name | Urban/rural classification[1] | Average population[2] | Population density (persons/km²) | Total cases (2001-2016) | Mean raw annual incidence rate[*] | Mean smoothed annual incidence rate[*] |
|---|---|---|---|---|---|---|---|
| Noosa Shire | Kin Kin | Rural Balance | 670 | 7 | 19 | 177 | 183 |
| Brisbane City | Fairfield | Major Urban | 859 | 725 | 24 | 175 | 139 |
| Scenic Rim Region | Roadvale | Rural Balance | 251 | 14 | 7 | 175 | 99 |
| Scenic Rim Region | Harrisville | Rural Balance | 537 | 17 | 15 | 174 | 132 |
| Brisbane City | Pinkenba | Major Urban | 323 | 23 | 9 | 174 | 0 |
| Sunshine Coast Region | Doonan | Other Urban | 3,032 | 94 | 84 | 173 | 168 |
| Sunshine Coast Region | Coochin Creek | Rural Balance | 74 | 1 | 2 | 170 | 95 |
| Scenic Rim Region | Kalbar | Rural Balance | 958 | 30 | 26 | 170 | 155 |
| Scenic Rim Region | Canungra | Rural Balance | 1,077 | 36 | 29 | 168 | 153 |
| Scenic Rim Region | Innisplain | Rural Balance | 75 | 3 | 2 | 168 | 57 |
| Ipswich City | Purga | Rural Balance | 505 | 9 | 13 | 161 | 154 |
| Scenic Rim Region | Mount Walker | Rural Balance | 117 | 4 | 3 | 160 | 93 |
| Gold Coast City | Jacobs Well | Other Urban | 1,612 | 119 | 41 | 159 | 149 |
| Brisbane City | Kooringal | Rural Balance | 39 | 249 | 1 | 158 | 72 |
| Moreton Bay Region | Closeburn | Rural Balance | 521 | 44 | 13 | 156 | 106 |
| Scenic Rim Region | Moorang | Rural Balance | 40 | 1 | 1 | 155 | 88 |
| Scenic Rim Region | Coleyville | Rural Balance | 162 | 4 | 4 | 154 | 135 |
| Moreton Bay Region | Laceys Creek | Rural Balance | 244 | 3 | 6 | 154 | 109 |
| Redland City | Russell Island | Other Urban | 2,486 | 142 | 61 | 153 | 0 |
| Sunshine Coast Region | Eudlo | Rural Balance | 979 | 49 | 24 | 153 | 113 |
| Moreton Bay Region | Mount Pleasant | Rural Balance | 291 | 8 | 7 | 150 | 116 |
| Sunshine Coast Region | Verrierdale | Rural Balance | 679 | 23 | 16 | 147 | 139 |
| Moreton Bay Region | Ocean View | Rural Balance | 820 | 21 | 19 | 145 | 99 |

[1] Based on ASGS classification of urban and rural, available from the Australian Bureau of Statistics.
[2] Based on census data, available from the Australian Bureau of Statistics.
[*] per 100,000 population
