# Supplementary material for: Spatial and temporal patterns of Ross River virus in south east Queensland, Australia: identification of hot spots at the rural-urban interface
Source: BMC Infect Dis. 2020 Oct 2;20:722. doi: 10.1186/s12879-020-05411-x (PMC7530966; doi:10.1186/s12879-020-05411-x)
Supplement: Supplementary file 7 — Additional file 7: Table S7. Summary of 45 persistent hot spots identified in both raw and smoothed incidence analyses from 2001 to 2016. [file 12879_2020_5411_MOESM7_ESM.pdf]

Table S7. Summary of 45 persistent hot spots identified in both raw and smoothed incidence analyses from 2001-2016.

LGA name	SSC name	Urban/rural category	Total cases	2001	2002	2003	2004	2005	2006	2007	2008	2009	2010	2011	2012	2013	2014	2015	2016	Total hot spot years
Sunshine Coast Region	Doonan	Other Urban	84	0	1	1	0	1	0	0	0	1	1	0	1	0	1	0	0	**7**
Noosa Shire	Tewantin	Major Urban	167	0	0	1	0	1	0	0	1	0	1	0	0	0	1	0	0	**5**
Noosa Shire	Cooroy	Rural Balance	142	0	0	1	0	0	0	0	0	1	0	0	1	1	1	0	0	**5**
Noosa Shire	Noosaville	Major Urban	98	0	0	1	0	1	0	0	0	1	1	0	0	0	1	0	0	**5**
Sunshine Coast Region	Eumundi	Rural Balance	75	0	0	1	0	1	0	0	1	0	0	0	0	0	1	0	1	**5**
Noosa Shire	Cooroibah	Rural Balance	40	0	0	1	0	1	0	0	0	0	1	0	0	1	1	0	0	**5**
Noosa Shire	Boreen Point	Rural Balance	19	0	0	1	0	1	0	0	0	0	1	0	1	0	0	0	1	**5**
Sunshine Coast Region	Verrierdale	Rural Balance	16	0	0	1	0	1	0	1	0	0	0	0	0	0	1	0	1	**5**
Noosa Shire	Cootharaba	Rural Balance	15	0	0	1	0	0	0	0	0	1	1	0	1	0	1	0	0	**5**
Sunshine Coast Region	Yandina Creek	Rural Balance	14	0	0	0	0	0	0	1	1	0	1	0	0	0	1	0	1	**5**
Noosa Shire	Pomona	Rural Balance	98	0	0	1	0	0	0	0	0	0	0	0	1	1	1	0	0	**4**
Sunshine Coast Region	Beerwah	Other Urban	88	0	0	1	0	0	0	0	0	0	1	0	0	1	0	0	1	**4**
Noosa Shire	Cooran	Rural Balance	56	0	1	1	0	0	0	0	0	0	0	0	1	0	1	0	0	**4**
Sunshine Coast Region	Mooloolah Valley	Other Urban	41	0	0	1	0	0	0	1	0	0	0	0	0	1	0	0	1	**4**
Sunshine Coast Region	Peregian Beach	Major Urban	40	0	0	1	0	0	0	0	0	1	0	0	0	0	1	0	1	**4**
Sunshine Coast Region	Conondale	Rural Balance	26	0	1	0	1	0	0	0	1	0	0	0	1	0	0	0	0	**4**
Noosa Shire	Lake Macdonald	Rural Balance	19	0	0	1	0	0	0	0	0	0	0	0	1	1	1	0	0	**4**
Sunshine Coast Region	North Arm	Rural Balance	11	0	0	1	0	0	0	1	0	0	0	0	0	0	1	0	1	**4**
Noosa Shire	Noosa North Shore	Rural Balance	9	0	0	1	0	1	0	0	0	0	1	0	0	0	1	0	0	**4**
Noosa Shire	Noosa Heads	Major Urban	78	0	0	1	0	1	0	0	0	0	0	0	0	0	1	0	0	**3**
Sunshine Coast Region	Glass House Mountains	Other Urban	76	0	0	0	0	0	0	0	1	0	0	0	0	1	0	0	1	**3**
Sunshine Coast Region	Landsborough	Other Urban	68	0	0	0	0	0	0	0	0	0	0	0	0	1	1	0	1	**3**
Moreton Bay Region	Elimbah	Other Urban	39	1	0	0	0	1	0	0	0	0	0	0	0	1	0	0	0	**3**

LGA name	SSC name	Urban/rural category	Total cases	2001	2002	2003	2004	2005	2006	2007	2008	2009	2010	2011	2012	2013	2014	2015	2016	Total hot spot years
Moreton Bay Region	Dayboro	Other Urban	34	0	0	1	0	0	0	0	0	0	0	0	0	1	0	1	0	**3**
Sunshine Coast Region	Maroochy River	Other Urban	21	0	0	1	0	0	0	0	0	0	0	0	0	0	1	0	1	**3**
Noosa Shire	Kin Kin	Rural Balance	19	0	0	1	0	0	0	0	0	0	0	0	1	0	1	0	0	**3**
Sunshine Coast Region	Ninderry	Rural Balance	14	0	0	0	0	0	0	1	1	0	0	0	0	0	1	0	0	**3**
Sunshine Coast Region	Beerburrum	Rural Balance	10	1	0	0	0	0	0	0	1	0	0	0	0	1	0	0	0	**3**
Ipswich City	Willowbank	Rural Balance	9	0	0	0	0	0	0	0	1	1	0	0	0	0	0	1	0	**3**
Sunshine Coast Region	Valdora	Rural Balance	6	0	0	0	0	0	0	0	1	0	0	0	0	0	1	0	1	**3**
Noosa Shire	Pinbarren	Rural Balance	5	0	0	1	0	0	0	0	0	0	0	0	1	0	1	0	0	**3**
Sunshine Coast Region	Coolum Beach	Other Urban	111	0	0	0	0	0	0	0	0	0	0	0	0	0	1	0	1	**2**
Gold Coast City	Ormeau	Major Urban	90	1	0	0	0	0	0	1	0	0	0	0	0	0	0	0	0	**2**
Sunshine Coast Region	Yandina	Other Urban	72	0	1	0	0	0	0	0	0	0	0	0	0	0	1	0	0	**2**
Gold Coast City	Jacobs Well	Other Urban	41	0	0	0	0	0	0	1	0	0	0	1	0	0	0	0	0	**2**
Sunshine Coast Region	Mount Coolum	Other Urban	31	0	1	0	0	0	0	0	0	0	0	0	0	0	1	0	0	**2**
Sunshine Coast Region	Peregian Springs	Major Urban	20	0	0	0	0	0	0	0	0	0	0	0	0	0	1	0	1	**2**
Scenic Rim	Harrisville	Rural Balance	15	0	0	0	0	0	1	0	0	0	0	0	0	0	0	1	0	**2**
Noosa Shire	Tinbeerwah	Rural Balance	15	0	0	1	0	0	0	0	0	0	0	0	0	0	1	0	0	**2**
Ipswich City	Purga	Rural Balance	13	0	0	0	0	0	0	0	0	0	0	0	1	0	0	1	0	**2**
Moreton Bay Region	Donnybrook	Rural Balance	12	1	0	0	0	1	0	0	0	0	0	0	0	0	0	0	0	**2**
Noosa Shire	Black Mountain	Rural Balance	10	0	0	0	0	0	0	0	0	0	0	0	0	1	1	0	0	**2**
Ipswich City	Walloon	Rural Balance	7	0	0	0	0	0	0	0	1	0	1	0	0	0	0	0	0	**2**
Sunshine Coast Region	Mount Mellum	Rural Balance	6	0	0	1	0	0	0	0	0	0	0	0	0	0	0	0	1	**2**
Sunshine Coast Region	Weyba Downs	Major Urban	5	0	0	0	0	0	0	0	0	1	0	0	0	0	1	0	0	**2**
**Total**			**1885**	**4**	**5**	**24**	**1**	**11**	**1**	**7**	**10**	**7**	**10**	**1**	**11**	**12**	**29**	**4**	**15**	
